# A Comparative Study Between Stapler Hemorrhoidopexy and Conventional Hemorrhoidectomy

**DOI:** 10.7759/cureus.102285

**Published:** 2026-01-25

**Authors:** Susmitha K Reddy, Vikram Sindagikar, Mallikarjun B Patil, Dayanand Biradar, Anand Suntan, Veena Korishetty

**Affiliations:** 1 General Surgery, Shri B. M. Patil Medical College, Hospital and Research Centre, BLDE (Deemed to be University), Vijayapura, IND

**Keywords:** conventional hemorrhoidectomy, hemorrhoids, hospital stay, postoperative pain, stapled hemorrhoidopexy

## Abstract

Background: Hemorrhoids are common anorectal disorders that frequently require surgical intervention. Conventional hemorrhoidectomy is effective but often associated with significant postoperative pain and prolonged recovery. Stapled hemorrhoidopexy has emerged as a less invasive alternative aimed at improving postoperative outcomes.

Objectives: To compare stapled hemorrhoidopexy and conventional hemorrhoidectomy with respect to operative parameters, postoperative pain, analgesic requirement, hospital stay, and postoperative complications.

Methods: This prospective comparative study was conducted in 50 patients with Grade II and III hemorrhoids who were divided into two groups: stapled hemorrhoidopexy (n = 25) and conventional hemorrhoidectomy (n = 25). All patients underwent standardized preoperative evaluation and surgery under spinal anesthesia. Operative duration, postoperative pain (visual analogue scale (VAS) score), duration of hospital stay, and complications were recorded.

Results: Baseline demographic and clinical characteristics were similar in the groups. Operative time was significantly shorter in the stapled group, with all procedures completed within 20 minutes, compared to longer durations in the conventional group (p < 0.001). VAS scores were significantly lower in the stapled group (2.40 ± 0.58 vs. 4.72 ± 0.46; p < 0.001), with reduced analgesic requirement. Hospital stay was significantly shorter following stapled hemorrhoidopexy (1.40 ± 0.58 vs. 2.38 ± 1.14 days; p < 0.001). Postoperative complications were more frequent in the conventional group, though differences were not statistically significant.

Conclusion: Stapled hemorrhoidopexy offers significant advantages over conventional hemorrhoidectomy in terms of operative efficiency, postoperative pain, analgesic requirement, and hospital stay, with comparable safety in patients with Grade II and III hemorrhoids.

## Introduction

Hemorrhoids are normal vascular cushions within the anal canal that play an important role in maintaining continence [1,2]. They consist of arterioles, venules, arteriovenous connections, smooth muscle fibers, and connective tissue embedded in the submucosa [1,2]. Based on their relationship to the dentate line, hemorrhoids are classified as internal or external. Internal hemorrhoids arise above the dentate line and are lined by columnar or transitional epithelium, whereas external hemorrhoids are located below the dentate line and are covered by squamous epithelium [3]. Internal hemorrhoids typically occur at the 3, 7, and 11 o’clock positions within the anal canal and are graded according to the extent of prolapse using Goligher’s classification [4].

The development of hemorrhoidal disease is multifactorial. Chronic constipation, prolonged straining during defecation, increased intra-abdominal pressure, and age-related degeneration of the supporting connective tissues are recognized contributing factors [1,5]. Clinically, patients commonly present with painless bright red rectal bleeding, prolapse, mucous discharge, and perianal discomfort. Chronic perianal irritation may lead to pruritus ani and adversely affect quality of life [6].

Management strategies for hemorrhoids depend on disease severity and symptom burden. Conservative measures and minimally invasive procedures are generally effective for early-stage disease, whereas patients with advanced or refractory hemorrhoids often require surgical intervention [7,8]. Conventional excisional hemorrhoidectomy, such as the Milligan-Morgan technique, remains the gold standard for advanced hemorrhoids because of its durability and low recurrence rates [9]. However, it is frequently associated with significant postoperative pain, prolonged recovery, and delayed return to normal activities [10].

Stapled hemorrhoidopexy was introduced as an alternative surgical technique aimed at reducing postoperative pain and improving early recovery [11]. The procedure involves circumferential resection of prolapsed rectal mucosa above the hemorrhoidal cushions, resulting in repositioning of the anal cushions and interruption of blood flow to the hemorrhoidal plexus while preserving the sensitive anoderm [12]. Several studies have demonstrated that stapled hemorrhoidopexy is associated with shorter operative time, reduced postoperative pain, shorter hospital stay, and earlier return to daily activities compared with conventional hemorrhoidectomy [13-15]. However, outcomes may vary depending on patient selection, surgical expertise, and healthcare settings, and concerns regarding long-term recurrence and increased procedural cost remain.

Given these considerations and the limited prospective data from many regions, the present study was undertaken to compare stapled hemorrhoidopexy and conventional hemorrhoidectomy in patients with Grade II and III hemorrhoids. The study aimed to evaluate operative parameters, postoperative pain, analgesic requirements, duration of hospital stay, and early postoperative complications to provide a balanced assessment of short-term outcomes associated with both procedures.

## Materials and methods

This prospective comparative observational study was conducted in the Department of General Surgery at Shri B. M. Patil Medical College Hospital and Research Centre, Vijayapura, Karnataka, India, from March 2024 to September 2025. Approval was obtained from the Institutional Ethics Committee (IEC approval number: BLDE (DU)/IEC-SBMPMC/002/2023-24).

Patients presenting with symptomatic Grade II or Grade III internal haemorrhoids refractory to conservative or minimally invasive treatment, classified according to Goligher’s classification [16], were enrolled. Grade II haemorrhoids were included only if symptoms persisted despite prior non-surgical management. Patients with acute thrombosed haemorrhoids were excluded to avoid confounding due to acute inflammation, emergency presentation, and differing pain profiles, as well as those with associated anal pathologies such as fistula-in-ano, anal fissure, or anal canal stenosis. Demographic, clinical, and perioperative data were collected prospectively for all participants.

The sample size was calculated using G*Power software version 3.1.9.7 [17], employing an independent samples t-test, based on operative duration reported by Chhikara et al. [18] (mean duration of surgery: 34.75 ± 16.5 minutes for conventional hemorrhoidectomy and 30.0 ± 9.03 minutes for stapled hemorrhoidopexy). With a power of 80%, a two-sided alpha error of 5%, and equal allocation between groups, 25 patients per group were required, resulting in a total sample size of 50 patients.

Patients were allocated into two equal groups (25 patients per group) based on surgeon preference after informed discussion with the patient, following sequential enrollment. Randomization was not performed due to ethical and practical constraints, including patient preference and institutional practice patterns. To reduce selection bias, prospective enrollment, standardized perioperative protocols, and objective outcome assessment were employed.

Group 1 included patients undergoing stapled hemorrhoidopexy using Longo's technique [19], while Group 2 included patients undergoing conventional open hemorrhoidectomy, as described by Milligan et al. [20]. All participants underwent standardized preoperative evaluation, including complete blood count, blood glucose, renal and liver function tests, coagulation profile, viral markers, electrocardiogram, chest radiography, and pre-anaesthetic fitness assessment.

All surgeries were performed under spinal anaesthesia by experienced surgeons following strict aseptic precautions. Operative duration was measured from completion of painting and draping to completion of the surgical procedure. Postoperatively, all patients received standardized care including antibiotics, sitz baths, and stool softeners as per institutional protocol. Postoperative urinary retention was monitored clinically and defined as the inability to void, requiring catheterization. Patients were monitored during hospital stay for postoperative pain, bleeding, urinary retention, wound healing, and duration of hospital stay.

Postoperative analgesia was standardized for all patients and was measured using the visual analogue scale (VAS), a validated and widely used pain assessment tool that is free for clinical and research use [21]. Intravenous paracetamol (1 g every eight hours) was administered during the first 24 hours, followed by oral paracetamol (650 mg every eight hours). Rescue analgesia in the form of intramuscular diclofenac (75 mg) was given as required for breakthrough pain. Analgesic requirement was recorded for each patient.

Patients were monitored daily during hospital stay for pain, bleeding, urinary retention, and wound-related complications. Planned outpatient follow-up visit was scheduled at one week postoperatively. Long-term follow-up for recurrence and late complications was not included in the study protocol.

Data were analyzed using Statistical Product and Service Solutions (SPSS, version 26; IBM SPSS Statistics for Windows, Armonk, NY). Normality of continuous variables was assessed using the Shapiro-Wilk test. Continuous variables were expressed as mean ± standard deviation and analyzed using an independent samples t-test or a Mann-Whitney U test, as appropriate. Categorical variables were expressed as frequencies and percentages and analyzed using the chi-square test or Fisher's exact test. No missing data were encountered, and analysis was performed on a per-protocol basis. A p-value <0.05 was considered statistically significant.

## Results

Both groups were comparable with respect to age distribution, gender, and grade of haemorrhoids. The majority of patients in both groups belonged to the 18-30 and >60 years age categories. Males predominated in both the stapler hemorrhoidopexy group 21 (84.0%) and the conventional hemorrhoidectomy group 24 (96.0%). Grade III haemorrhoids were slightly more common than Grade II in both groups (Table 1).

**Table 1:** Baseline characteristics (n = 50) This table compares the baseline demographic characteristics and clinical severity of haemorrhoids [16] between patients undergoing stapler hemorrhoidopexy (Group 1) and conventional hemorrhoidectomy (Group 2). Categorical variables are expressed as frequency and percentage. Intergroup comparisons were performed using the chi-square test or Fisher's exact test, as appropriate. A p-value <0.05 was considered statistically significant.

Variable		Group 1 (Stapler Hemorrhoidopexy) (n=25)	Group 2 (Conventional Hemorrhoidectomy) (n=25)	Test statistic (df)	p-value
Age group (years)	18–30	7 (28.0%)	6 (24.0%)	χ² = 1.38 (df = 4)	0.848
	31–40	4 (16.0%)	7 (28.0%)		
	41–50	5 (20.0%)	6 (24.0%)		
	51–60	3 (12.0%)	2 (8.0%)		
	>60	6 (24.0%)	4 (16.0%)		
Gender	Male	21 (84.0%)	24 (96.0%)	χ² = 0.88 (df = 1)	0.349
	Female	4 (16.0%)	1 (4.0%)		
Grade of haemorrhoids	Grade II	11 (44.0%)	12 (48.0%)	χ² = 0.08 (df = 1)	0.776
	Grade III	14 (56.0%)	13 (52.0%)		

All patients in the stapler hemorrhoidopexy group underwent surgery within 20 minutes, whereas all conventional hemorrhoidectomy procedures required 20 minutes or longer, a difference that was statistically significant (p < 0.001). Postoperative pain was significantly lower in the stapler group. The mean pain score was markedly less in the stapler group (2.40 ± 0.58) than in the conventional group (4.72 ± 0.46). Correspondingly, analgesic requirement was significantly reduced in the stapler group, with only five (20.0%) requiring analgesics compared to 25 (100.0%) in the conventional group (p < 0.001) (Table 2).

**Table 2:** Operative parameters and postoperative pain comparison This table summarizes operative duration, postoperative pain scores [21], and analgesic requirements between the two surgical groups. Duration of surgery and pain categories are expressed as frequencies and percentages, while pain scores are presented as mean ± standard deviation. Categorical variables were compared using the chi-square test, and continuous variables were compared using the Independent samples t-test. A p-value <0.05 was considered statistically significant.

Parameter		Group 1 (Stapler Hemorrhoidopexy) (n=25)	Group 2 (Conventional Hemorrhoidectomy) (n=25)	Test statistic (df)	p-value
Duration of surgery	<20 minutes	25 (100.0%)	0 (0.0%)	χ² = 50.0 (df = 1)	<0.001
	≥20 minutes	0 (0.0%)	25 (100.0%)		
Pain score (VAS)	≤3	24 (96.0%)	0 (0.0%)	χ² = 46.1 (df = 1)	<0.001
	≥4	1 (4.0%)	25 (100.0%)		
	Mean ± SD	2.40 ± 0.58	4.72 ± 0.46		
Analgesic requirement	Yes	5 (20.0%)	25 (100.0%)	χ² = 33.3 (df = 1)	<0.001

A significantly higher proportion of patients in the stapler hemorrhoidopexy group 24 (96.0%) were discharged within two days, whereas the majority of patients in the conventional hemorrhoidectomy group 24 (96.0%) required hospitalisation for three days or more. The mean duration of hospital stay was significantly shorter in the stapler group (1.40 ± 0.58 days) compared to the conventional group (2.38 ± 1.14 days), and this difference was statistically significant (p < 0.001) (Table 3).

**Table 3:** Hospital stay This table depicts the duration of postoperative hospital stay among patients undergoing stapler hemorrhoidopexy and conventional hemorrhoidectomy. Hospital stay is expressed as frequency and percentage, and mean duration is presented as mean ± standard deviation. Categorical variables were analyzed using the chi-square test, and mean hospital stay was compared using the independent samples t-test. A p-value <0.05 was considered statistically significant.

Hospital stay (days)	Stapler Hemorrhoidopexy (n=25)	Conventional Hemorrhoidectomy (n=25)	Test statistic (df)	p-value
≤2 days	24 (96.0%)	1 (4.0%)	χ² = 44.1 (df = 1)	<0.001
≥3 days	1 (4.0%)	24 (96.0%)		
Mean ± SD	1.40 ± 0.58	2.38 ± 1.14		

Postoperative complications were more frequent in the conventional group, but the difference was not statistically significant. Postoperative bleeding was predominantly mild and self-limited, with only one patient in the conventional hemorrhoidectomy group requiring local intervention, and no cases requiring blood transfusion or reoperation. Urinary retention occurred only in patients undergoing conventional hemorrhoidectomy; all affected patients had received spinal anesthesia and were managed conservatively with temporary catheterization (Table 4).

**Table 4:** Postoperative complications This table compares the incidence of postoperative complications between the two surgical groups. Data are presented as frequencies and percentages. Intergroup comparisons were performed using Fisher's exact test due to small cell counts. A p-value <0.05 was considered statistically significant.

Complication	Stapler Hemorrhoidopexy (n=25)	Conventional Hemorrhoidectomy (n=25)	Fisher’s exact p-value
Any bleeding	3 (12.0%)	7 (28.0%)	0.157
Surgical site infection	0 (0.0%)	3 (12.0%)	0.235
Urinary retention	0 (0.0%)	3 (12.0%)	0.235
Anal incontinence (flatus)	0 (0.0%)	3 (12.0%)	0.235
Overall complication rate	3 (12.0%)	16 (64.0%)	0.0001

## Discussion

This study compared stapled hemorrhoidopexy and conventional hemorrhoidectomy in patients with Grade II and III hemorrhoids, with emphasis on operative efficiency, postoperative recovery, and early complications. Baseline demographic characteristics and hemorrhoid grade were comparable between the two groups, reducing the likelihood that outcome differences were related to baseline imbalance. Similar demographic comparability has been reported by Agrawal et al. and Sachin et al., supporting valid intergroup comparison [13,14].

Operative duration was significantly shorter in the stapled hemorrhoidopexy group. All stapled procedures were completed within 20 minutes, whereas conventional hemorrhoidectomy required longer operative times. Operative time was analyzed categorically to reflect workflow efficiency in our institutional setting. Prior studies reporting mean operative times provide a supportive context. Agrawal et al. reported mean operative times of 22.27 minutes for stapled hemorrhoidopexy and 25.00 minutes for conventional hemorrhoidectomy (p < 0.001) [13]. Ram et al. demonstrated a larger difference, with stapled procedures averaging 24.28 minutes compared with 45.41 minutes for conventional surgery [15]. Ali et al. also reported shorter operative duration with stapled hemorrhoidopexy (31.53 ± 5.29 vs. 35.73 ± 8.15 minutes; p = 0.008) [22]. These findings collectively support the operative efficiency of the stapled technique.

Postoperative pain outcomes favored stapled hemorrhoidopexy in the present study. Patients in the stapled group reported significantly lower VAS scores at 24 hours and required fewer rescue analgesics. These results are consistent with those of Sachin et al., who reported lower postoperative pain scores in stapled hemorrhoidopexy patients [14]. Agrawal et al. demonstrated significantly reduced pain scores on postoperative days one, three, and seven following stapled hemorrhoidopexy [13]. Shah et al. also observed that some patients undergoing stapled hemorrhoidopexy required no postoperative analgesia, whereas analgesic use was universal after conventional hemorrhoidectomy [23]. Although pain assessment time points varied across studies, the consistent trend favors stapled hemorrhoidopexy for early postoperative pain control.

Hospital stay was significantly shorter in the stapled hemorrhoidopexy group, indicating faster postoperative recovery. Agrawal et al. reported mean hospital stays of 1.53 ± 0.51 days for stapled hemorrhoidopexy and 2.77 ± 0.77 days for conventional hemorrhoidectomy (p < 0.001) [13]. Sachin et al. similarly noted earlier discharge in stapled patients [14]. Ali et al. also demonstrated reduced hospitalization following stapled hemorrhoidopexy [22]. In the present study, longer hospital stay in the conventional group was primarily related to higher postoperative pain, need for parenteral analgesia, and monitoring for complications such as urinary retention following spinal anesthesia.

Postoperative complications were numerically more frequent in the conventional hemorrhoidectomy group, although the differences were not statistically significant. Bleeding was generally mild and self-limiting, with only one patient requiring local intervention. Similar trends toward higher complication rates after conventional hemorrhoidectomy have been reported previously. Yadav et al. observed higher postoperative infection rates in conventional hemorrhoidectomy compared with stapled hemorrhoidopexy (35% vs. 5%; p = 0.018) [24]. Manzoor et al. also reported urinary retention and delayed wound healing more frequently following conventional hemorrhoidectomy [25]. These findings suggest that both procedures are safe, with stapled hemorrhoidopexy demonstrating fewer early postoperative complications in selected patients.

It is important to acknowledge that stapled hemorrhoidopexy has been associated with higher long-term recurrence rates and increased procedural costs compared with conventional hemorrhoidectomy. These outcomes were not evaluated in the present study, as follow-up was limited to the early postoperative period.

Alternative treatment modalities, such as rubber band ligation, transanal hemorrhoidal dearterialization, and laser-based procedures, play an important role in the management of hemorrhoidal disease in appropriately selected patients. Rubber band ligation is widely used for Grade II and selected Grade III hemorrhoids, particularly in patients with predominant bleeding and minimal prolapse. Transanal hemorrhoidal dearterialization and laser techniques aim to reduce arterial inflow to the hemorrhoidal plexus while minimizing tissue trauma, which may result in reduced postoperative pain and faster recovery. However, symptom control with these techniques can be variable, and repeat interventions may be required, especially in patients with significant mucosal prolapse. Stapled hemorrhoidopexy, by repositioning prolapsed mucosa and interrupting blood supply circumferentially, offers a surgical option that addresses prolapse more directly while maintaining favorable short-term postoperative outcomes [26].

The strengths of this study include its prospective design, standardized perioperative care, and objective assessment of operative and early postoperative outcomes. However, limitations include the small sample size, single-center design, non-randomized allocation, and lack of long-term follow-up. Larger randomized studies with extended follow-up and cost analysis are required to better define the optimal surgical approach for hemorrhoidal disease.

## Conclusions

In this prospective comparative study, stapled hemorrhoidopexy demonstrated advantages in short-term postoperative pain control and length of hospital stay when compared with conventional hemorrhoidectomy. Operative time and analgesic requirement were also lower in the stapled group in this cohort. Although postoperative complications were numerically more frequent following conventional hemorrhoidectomy, these differences were not statistically significant. Larger randomized studies with longer follow-up are required to evaluate long-term outcomes, including recurrence and late complications, and to further define the role of stapled hemorrhoidopexy in hemorrhoid management.
